# 

**Published:** 1852-01

**Authors:** 


### INDEX.

A

Abdominal tumors interfering with pregnancy 546
Abscess of lung following pueumonia 463
Academy of Medicine of Paris 117, 670
Acetate of lead producing colica pictonum 60
Action of water on lead pipes 56
Action of medicines 130
Action of chloroform 548
Action of cold, warmth, and light upon crystalline lens 553
Action of cold upon coagulability of blood 559
Action of poisons on the nervous system 698
Acute metritis treated successfully with urate of ammonia 531
Adhesive plaster as a means of making extension in fractures of lower extremities 685
Adhesive plaster, as a valuable constituent in the composition of various apparatus used in surgery. By D. Gilbert, M. D. 784
Air passages, a treatise on diseases of, &c. &c., by H. Green, M.D., bib. notice of 662
Air passages, case of foreign body in 192
Albuminous urine, causes of 201
Alumina, tannate of 119
American Medical Association, organizing of 137
American Medical Association, transactions of, bib. notice of 93
American Medical Association, delegates to 265
American Medical Society in Paris 184
Ammonia, sesquicarbonate of, in lepra and psoriasis 124
Amorphous phosphorus 860
Amputation of shoulder-joint 468
Anal region, prurigo of 120
Analysis of physiology; being a condensed view of the most important facts and doctrines, &c. By Jno. J. Reese, M. D., &c., bib. notice of 535
Analytical compendium of the various branches of medical science. By Jno. Neill, M. D., and F. G. Smith, M. D., &c., bib. notice of 48
Anatomy 124, 203, 479
Anatomical investigations in the elucidation of cutaneous diseases. By G. Simon, M. D., &c., bib. notice of 170
Anæsthesia, discussion on, at Philadelphia County Medical Society 243, 298
Anæsthesia, means of measuring degrees of 503
Anæsthetic action of ice 583
Aneurism, novel treatment of 412
Aneurism by anastomosis 454
Animal graft, singular case of 560
Animals deprived of medulla oblongata, persistence of life in 565
Annual report of Committee on Public Hygiene, by W. Jewell, M. D. 1
Announcement of a new Medical School in Cincinnati 593
Antidote for cupreous salts 614
Antidote in poisoning with arsenic 515
Anterior inferior spinous process of ilium, fracture of 159
Antiseptic employment of sulphate of zinc 203
Answer to a friend, on organizing the American Medical Association 527
Appearance of menses, conception before 581

Appointment 748
" in University of N. Y. 265
" of Dr. J. Neill 391
" in University of Louisville 593
" in University of Pennsylvania, 593
" of Dr. Leidy 537
Army, medical department of 184
Arsenical poisoning 115
Arsenic eaters 190
Articular inflammation treated with urate of ammonia 69
Asthma, spasmodic, case of 23
Autobiography of Berzelius 750

B

Balsam of Peru, extraction of 118
Barium, use of, in Pharmacy 548
Barnard Holts' stricture dilator 611
Baron Humboldt 749
Basis of Eau Medicinale d'Husson 450
Bedford Springs 358
Bed-sore successfully treated by a galvanic current 25
Berberine, sulphate of 277
Bell, Dr. John, resignation of 268
Bennett on molecular origin of tissues 480
Bibliographical Notices, 35, 83, 170, 260, 331, 435, 533, 643, 723, 802
Bivalve speculum, modification of 567
Blood, action of cold on coagulability of 559
Board of Naval Surgeons 1[illegible]5
Bond, T. E., a practical treatise on dental medicine, by 805
Brain, compression of, relieved by trephining 64
Brain, softening of, after ligation of carotid artery 122
Brazen Homœopath 188
Brazil, sketches of, including new views on Tropical and European fever, &c. By R. Dundas, M. D., bib. notice of 656
British American Med. and Phys. Journal 267
Bright's disease, remarks on microscopic characters of urine, in 466
Bronchophony, mechanism of 456
Brown-Sequard's lectures 391
Brown-Sequard, Dr., lectures, notice of 808
Burman Empire, epidemic diseases of 282

C

Cadaveric rigidity, experiments on 124
Cadmium, use of, in pharmacy 548
Cancer of skin, remarks on 229
Carotid artery, effects of ligation of 122
Case of spasmodic asthma 23
Case of bed-sore successfully treated by a galvanic current 23
Cases of articular inflammation treated with urate of ammonia 69
Case of latent phthisis pulmonalis terminating suddenly in pneumothorax 273
Cases of epithelial tumors 287
Case of hydrophobia 464
Case of animal graft 560
Cases of tape worm 629
Case of difficult labor from deformity of fœtus 689
Cataract, structure of 198
Causes of torpidity of tenrec 549
Causes of albuminous urine 201
Cauterization of urethra 427
Central seat of general and tactile sensibility 626
Character of urine in Bright's disease 466
Charing Cross Hospital 468, 742
Charities, medical 188
Charleston Preparatory Medical School 181
Chemistry, manual of Physiological. By Prof. C. G. Lehmann, bib. notice of 35
Chemistry, physiological and pathological, manual of. Prepared by Dr. A. Moser and Dr. J. C. Strahl, bib. notice of 35
Chemistry in its relations to physiology, dietetics, agriculture, commerce, and political economy. By Justus Von Liebig, bib. notice of 35
Chemistry, elements of, including the application of the science in the arts. By Thos. Graham, F. R. S., &c. Edited, with notes, by R. Bridges, M. D., &c., bib. notice of 435
Chemistry 548, 613, 680
Chloroform successfully employed in strangulated crural hernia 280
Chloroform as an emmenagogue 425
" deaths from 59, 454
" action of 548
" in infantile convulsions and other spasmodic diseases 667
Cholera 748
" in military practice 504
Chronic ovarian tumors, diagnosis of 604
Circular of American Med. Society in Paris 184

Circulars, medical 467
Climate and epidemic diseases of Rangoon 282
Clinical reports, 162, 322, 383, 430, 586, 749, 790
Clinic of Jefferson Medical College, 162, 322, 383, 430, 586
Clinic of Pennsylvania College 749
Clinical reports on continued fever, &c. By A. Flint, M. D., &c., bib. notice of 731
Cocculus indicus, case of poisoning by 227
Colica pictonum 60
Compendium, analytical, of the various branches of medical science. By J. Neill, M. D. and F. G. Smith, M. D., &c. &c., bib. notice of 48
Complete treatise on midwifery; or the theory and practice of tokology, &c. &c. By Alf. A. L. M. Velpeau, M. D., &c., translated by C. D. Meigs, M. D., &c., with additions by W. Byrd Page, M. D., &c., bib. notice of 45
Compression of brain 64
Comparative value of cod liver oil, mixed with iodine 118
Concours for chair of hygiene in faculty of medicine of Paris 449
Conception before the appearance of the menses 581
Constriction of œsophagus 544
Constitutional and hereditary syphilis. By E. Wilson, F. R. S., &c., bib. notice of 723
Convulsive movements of stump 468
Convulsive affection produced by extensive injury of spinal cord 561
Corpus luteum, formation of, in cases of impregnation 203
Coryza, instantaneous cure of 546
Cost of doctorate in Paris 56
Cranium, fracture of 582
Crossed transmission of impressions in the spinal cord 703
Croup, operation of tracheotomy for relief of 222
Croup in the adult 544
Crystalline lens influenced by cold, warmth, and light 553
Cupreous salts, antidote for 614
Curious cutaneous disease 74
Curiosities of the microscope, &c. By Rev. J. A. Wythes, M. D., bib. notice of 733

D

Datura stramonium, poisoning by 199
Death of Dr. Leiper 50
" Dr. Hooper 58
" Priessnitz 116
" Dr. Jas. Arthur 114
" Dr. S. A. Drane 114
" Dr. Jas. Cameron 114
" Dr. P. M. Smith 110
" M. Gannal 189
" Baron Pasquier 188
" Dr. Grant 340
" Dr. John Dalrymple 450
" Dr. J. B. Rogers 537
" Dr. G. W. Patterson 593
" Dr. Isaac Parrish 593
" M. Dize 749
" Dr. Jonathan Cowdery 808
" Dr. Daniel Drake 808
Deaths from chloroform 59, 463
Deformity of fœtus causing difficult labor 687
Degrees of anæsthesia and hyperæsthesia 503
Delegates from Medical Society of Delaware 49
Delegates to American Medical Association 265
Delaware County Medical Society 265
Dentists, remarks and recommendations on the professional education of. By Jno. Trenor, M. D., bib. notice of 180
Dentists, professional education of 368
Diagnosis of chronic ovarian tumors 604
Diet in protracted fevers 122
Discussion on induction of premature labor 670
Diseases of skin elucidated by anatomical investigations. By G. Simon, M. D., &c., bib. notice of 180
Diseases of chest, treatise on. By Jno. A. Swett, M. D., &c., bib. notice of 181
Diseases in which a resort to the Bedford Springs is useful 358
Diphtheritis of the glands in some cases of paralysis 545
Diphtheritis, laryngo-bronchial, some remarks on 544
Disulphate of quinia, solution of, by tartaric acid 204
Doctorate in Paris 56
Donation, munificent 189
Drug-inspection law 663
Dysentery 570
Dysentery of Lancaster county 205

E

Eaters of arsenic 170
Eau medicinal d'Husson, basis of 450

Editorial, 49, 110, 181, 339, 391, 443, 536, 593, 663, 743, 808
Editorial courtesy 391
Education of Dentists 368
Effects of iodine on the glandular system 675
Election of Dr. E. Hartshorne 537
Election of officers of Northern Medical Association 183
Elements of chemistry, including the applications of the science in the arts, by Thos. Graham, F. R. S., &c. Edited, with notes, by R. Bridges, M. D., &c., bib. notice of 435
Elements of materia medica and therapeutics. By J. Pereira, M. D., F. R. S., &c., bib. notice of 44
Embryonic vertebrata, transportation of two aortæ into 204
Emigrants' hospital 748
Emmenagogue effects of chloroform 425
Employment of sulphate of zinc as an antiseptic 203
Endosmotic action of medicines 130
England, Royal Society of 114
Entropium proved to depend on muscular action 608
Epidemics, progress of 449, 666
Epidemic diseases of Rangoon 282
Epidemic jaundice 58
Epidemic cholera, a history of, at Chambersburg, Pa., during the summer of 1852. By A. H. Senseny, M. D. 774
[illegible]demilogical Society of London 115
Epithelial tumors, remarks on 229
Errata 746
Ergot of rye in treatment of paraplegia 121
Essays on life, sleep, pain, &c. By S. H. Dickson, M. D., bib. notice of 180
Esophagus, spasmodic constriction of 544
Esquimaux, parturition among 473
Etherization in parturition, nineteen centuries ago 267
Examination of the doctrines and evidences of homœopathy. By W. Hooker, M. D., bib. notice of 107
Examination of ointments containing oxide of mercury 615
Experimental researches applied to physiology and pathology, 481, 549, 617, 698
Experiments proving that life may be preserved in warm-blooded animals, after the destruction of a considerable part of the spinal marrow 321
Explanation 443
Extension in fractures of lower extremity effected by adhesive plaster 685
Extract from address of Dr. Samuel Grimes 445
Extracts from a lecture on the present position in Europe of some of the most interesting and important points in modern surgery 28, 76
Extract from a review of Dr. Gross' work on the urinary organs 536
Extraction of balsam of Peru 118
Extramural interments in London 185

F

Familiar lectures on chemistry, in its relations to physiology, dietetics, agriculture, commerce, and political economy. By Justus Von Liebig, bib. notice of 35
Fecundity of a free-martin 82
Fever, spread of, in Ireland 266
Fevers, protracted, diet in 122
Fœtus, influence of imagination of mother upon 344
Foreign body in knee joint 578
Foreign quackeries 663
Foreign body in larynx, operation for 215
Foreign body in air passages, tracheotomy performed for 192
Free hospital 743
Fracture of anterior inferior spinous process of ilium 159
Fractures of lower end of radius, and their management 146
Fracture of cranium 582
French revolution 116
French and American experiments on cadaveric rigidity 124
Functions of the great sympathetic nerve 413
Functions of organic life, influence of the nervous system upon 486

G

Galbanum plant 679
Galvanic current in treatment of bed-sore 25
Galvanic current employed in removal of nævus 197
Gazette, London Medical 116
Gaultheria, poisoning by oil of 347
Gaurana 616
Genital regions, prurigo of 120

Geology applied to the study of disease 19
George Ord, Esq. 58
Gilbert, D., on adhesive plaster as a valuable constituent in the composition of various apparatus used in surgery 784
Glandular system, effects of iodine on 675
Gobrecht's clinical reports 749, 790
Gonorrhœa 117
Gout, proximate cause and treatment of 595
Great sympathetic nerve, functions of 413
Guy's Hospital 743

H

Handbook, prescriber's complete, by Trousseau and Reveil 801
Heart, sounds of 135
Hereditary insanity 199
Hernia, strangulated, reduced during vomiting 202
Hernia, strangulated crural, successfully treated with chloroform 280
Hernia, strangulated, new mode of reducing 547
Homœopathy 111
Homœopathy; an examination of its doctrines and evidences. By W. Hooker, M. D., &c., bib. notice of 107
Honors of medical men 189
Horner, W. E., surgical sketches by 754
Hospital of Protestant Episcopal Church of Philadelphia 537
Hunterian School of Medicine 746
Hydatids of liver evacuated externally 120
Hydrated peroxides of iron and magnesia as antidotes in poisoning with arsenic 215
Hydrophobia, case of 464

I

Ice as a local anæsthetic 585
Iliac artery, primitive, ligation of 611
Illustrated manual of operative surgery and surgical anatomy. By MM. Bernard and Hunter, with notes and additions by W. H. Vanburen, M. D., and C. E. Isaacs, M. D., bib. notice of 260, 533
Impressions, crossed transmission of, in spinal cord 703
Infant, lactation in 50
Inanition, remarkable case of 268
Influence of imagination of mother upon the fœtus 344
Influence of nervous system upon functions of organic life 486
Influence of poisons upon animal heat as a cause of death 550
Influencing the general temperature by changing the temperature of one of the extremities 556
Infantile convulsions, chloroform in 667
Influence of temperature of a warm blooded animal upon the duration of its life, when asphyxiated 617
Injuries of spinal cord giving rise to convulsive affections 561
Injuries of nervous system producing turning and rolling phenomena 498
Instruction in microscopy 110
Insanity, hereditary 199
Instrument for cauterizing urethra 427
Interments in London 185
International sanitary convention in Paris 190
Intermittent coryza, instantaneous cure of 546
Inquiry into cause and treatment of gout 595
Iodine, effects of, on the glandular system 675
Iodine rendered soluble by syrup of orange peel and tannin 119
Iron, hydrated peroxide of, in poisoning with arsenic 515
Itch insect 266
Jefferson Medical College, Clinic of 162, 322, 383
Jerking movements of stump 458
Jewell on mortality of Philadelphia 350, 515, 689

K.

King's College Hospital 743
Kousso, properties of 675

L.

Labor, relaxation of symphysis pubis after 136
Lactation in the infant 50
Lancaster County, observations on epidemic dysentery of 205
Laryngo-bronchial diphtheritis, or croup in adults 544
Laryngotomy, successfully performed 215
Latent phthisis pulmonalis terminating suddenly in pneumothorax 273
Lawsuit of University of Louisville 268
Lead pipes, action of water on 56

Lead poisoning 679
Lectures on principles and practice of surgery. By B. B. Cooper, F. R. S., &c. bib. notice of 643
Lepra, sesquicarbonate of ammonia in 124
Letter from Dr. Hastings 594
Ligation of primitive iliac artery 611
Light, action of a crystallized lens 553
Liver, hydatids of, evacuated externally 120
London Hospital 463, 744
London Epidemilogical Society 115
London Medical Times & Gazette 116
Lucifer-match making 680
Lung, abscess of, following pneumonia 463

M.

Magnesia, hydrated peroxide of, an antidote in poisoning with arsenic 515
Management of fracture of lower end of radius 146
Man, temperature of 554
Manganese as an adjuvant to iron 750
Manual of physiological chemistry By Prof. C. G. Lehman, bib. notice of 35
Manual of physiological and pathological chemistry. By Dr. A. Moser and Dr. J. C. Stahl, bib. notice of 35
Manual of diseases of the skin, from the French of MM. Cazenave and Schedel, with notes and additions. By J. H. Burgess, M. D. and H. D. Buckley, M. D., &c. bib. notice of 170
Manual of operative surgery and surgical anatomy, illustrated. By MM. Bernard and Huette, with notes and additions by W. H. Vanburen, M. D., and C. E. Isaacs, M. D. bib. notice of 260
Materia Medica 118, 204, 450, 548, 616, 675, 750
Match-making 680
Meaning of term ulceration, as at present applied to uterine diseases 474
Measurement of degrees of anæsthesia and hyperæsthesia 503
Mechanism of bronchophony 456
Medical Society of Delaware 49
Medical News 55, 184, 264, 392, 345, 503, 537, 666, 747
Medical Times and Gazette 116
Medical Department of Army 184
Medical department U. S. Navy 808
Medical Charities 188
Medical men, honors of 189
Medical museums 189
Medical topography of Lancaster county 205
Medical circulars 267
Medical organization, remarks on 420
Medical student's vade mecum, a compendium of anatomy, physiology, &c. &c. By Geo. Mendenhall, M. D., bib. notice of 442
Medical Schools of Philadelphia 733
Medical Schools of London 734
Medical fees in Spain 749
Medicine, a treatise on practice of. By G. B. Wood, M. D. &c. bib. notice of 659
Medicines, endosmotic action of 130
Meigs' clinical reports 322, 383, 436, 586
Menses, conception after appearance of 581
Mercury, substitute for in syphylitic diseases 198
Mercury, test for presence of 614
Mercury, examination of ointments containing oxide of 615
Metritis, case of 531
Microscope, curiosities of. By Rev. J. A. Wythes, M. D. bib. notice of 733
Microscopy, instruction in 110
Microscopic anatomy of the human body in health and disease. By A. H. Hassall, M. D., &c., with additions by H. Vanarsdale, M. D., bib. notice of 105
Microscopic characters of the urine in Bright's disease 466
Microscopic examination of relaxed uvula 674
Middlesex hospital 744
Mineral springs 666
Mode of reducing strangulated hernia 547
Modification of bivalve speculum 367
Molecular origin of the tissues 480
Monument to Dr. Jenner 50
Monstrosity 203
Mortality of the States 268
Mortality of Philadelphia 350, 515, 689
Mummy found in St. Stephen's crypt 185
Munificent donation 189
Muscular contraction 124

N.

Nævus removed by heated platinum wire 197
Naval assistant surgeons 186
Naval surgeons 340
" board of 185

Nerves, outlines of, with short descriptions, designed for the use of medical students. By John Neill, M. D. bib. notice of 263
Nerve of organic life, functions of 413
Nervous system, reparative power of 497
Nerve-tubes, relation between the organization of, and their vital properties 563
New medical schools 748
New mode of reducing strangulated hernia 547
New instrument for cauterizing the urethra 427
New lights and new livers 269
New York, appointments in the University of 265
News, medical, 183, 392, 445, 593
Nickel, use of, in pharmacy 548
Normal temperature of man 554
Northern medical association 183
Note on sulphate of berberine 277
Notes of a case of latent phthis pulmonalis, terminating suddenly in pneumothorax 273
Notes on Bedford Springs 358
Notes from my case book 634
Novel treatment of aneurism 412
Number of physicians in Paris 412
Nutrition of muscles during their contraction 428

O.

Observations on sounds of heart 135
Observations on cholera in military practice 504
Obstetrics 186, 473, 546, 618, 647
Obstetrics, the science and the art of. By C. D. Meigs, M. D. &c. bib. notice of 441
Obstinate intermittent coryza 546
Occasional organic union of contiguous teeth 479
Oleum gaultheriæ, poisoning by 347
Operation, Taliacotian 341
Operative surgery, system of, based upon the practice of surgeons in the United States, &c. By H. H. Smith, M. D. bib. notice of 438
Opposition to vaccination 449
Orange colored sputa without pneumonia 671
Ord, Geo. Esq. 58
Organic life, functions of, influenced by nervous system 486
Organic life, nerve of 413
Organizing of American Medical Association 137
Origin of the tissues 405
Original communications 1, 69, 137, 273, 341, 413, 481, 549, 617, 753
Our Journal 49
Outlines of the nerves, with short descriptions, designed for the use of medical students. By John Neill, M. D., &c. bib. notice of 263
Ovarian tumors, diagnosis of 604
Oxygen, state of, in blood 613

P.

Paracentesis thoracis in pleurisy 603
Paralysis, diphtheritis of glands in 545
Paraplegia, successfully treated by ergot of rye 121
Paris, cost of the doctorate in 56
" annual public meeting of the Academy of 117
Parturition among the Esquimaux 473
Pasquier, death of 188
Pathological anatomy of spleen in the intermittent fever of Madagascar 545
Pathology 60, 120, 199, 454, 538, 595, 671
Pennsylvania Medical College 339, 708
Pennsylvania Hospital 184
" " for insane, report of for year 1851. By T. S. Kirkbride, M. D., bib. notice of 177
Persistence of life in animals deprived of a medulla oblongata 565
Persistence of life in frogs, after losing half the ventricle of the heart 559
Pessary, removal of, after forty-one years' residence in pelvis 616
Pharmaceutical uses of barium, cadmium and nickel 548
Phenomena of turning and rolling, produced by injuries of the nervous system 498
Philadelphia county Medical Society 50
Philadelphia, mortality of 350, 513, 689
Philadelphia College of Pharmacy 50
Phillips's review of Bernard's theory of an hepatico-renal circulation 787
Phosphorus, amorphous 680
Physicians' prescriptions 50, 265
" " in Paris 412
" visiting list, diary, &c. for 1853, bib. notice of 662
Physiology, analysis of, &c. By John J. Reese, M. D., bib. notice of 535
Physiology 124, 479, 595
Physiological chemistry, manual of. By Prof. C. G. Lehmann, bib. notice of 35

Physiological and pathological chemistry after the most recent sources. Prepared by Dr. A. Moser and Dr. J. C. Strahl, bib. notice of 35
Physiology and interchange of matter in plants and animals. By Dr. Jac. Moleschott, bib. notice of 35
Pneumothorax 273
Poisons, mode of action of, on nervous system 698
Poisoning by oleum gaultheriæ 347
Poisons causing death by influencing animal heat 550
Poisoning with arsenic, antidotes in 515
" by lead 679
Poisoning, arsenical 115
" by datura stramonium 199
" from external application of cocculus indicus 227
Practice of medicine 60, 120, 199, 454, 638, 671
" " treatise on. By G. B. Wood, M. D. &c. bib. notice of 659
Practical treatise on diseases of lungs and heart. By W. H. Walshe, M. D. bib. notice of 83
Practical treatise on dental medicine; by T. E. Bond, M. D. 805
Pregnancy complicated with abdominal tumors 546
Premature labor, induction of 670
Preparation of pure fatty acids for manufacture of candles 683
Preparatory medical school 181
Prescriber's complete handbook. By Trousseau and Reveil 802
Present to Royal College of Surgeons 449
Present position in Europe of some of the most interesting and important points of modern surgery 28
Preissnitz, death of 116
Primary medical school 56
Principles of surgery. By J. Miller, F. R. S. &c. Revised with additions by F. W. Sargent, M. D., bib. notice of 535
Principles and practice of surgery, lectures on. By B. B. Cooper, F. R. S. &c., bib. notice of 643
Principles and practice of surgery. By W. Pirrie, F. R. S., &c. Edited with additions by J. Neill, M. D., &c. bib. notice of 643
Proceedings of Pennsylvania State Medical Society 398
Professional education of dentists 368
Professional appointments 748
Progress of epidemics 449, 666
Properties of kousso 675
Protracted fevers, diet in 122
Proximate cause of gout 595
Prurigo of genital and anal regions 120
Prune-juice and orange-colored sputa without pneumonia 671
Public hygiene, annual report of committee on 1
Pulmonarp emphysema 538

Q.

Quackeries, foreign 663
Quiniæ disulphas, solution of by tartaric acid 204

R.

Radical cure of reducible hernia, committee on 58
Record of medical science 60, 117, 192, 271, 595, 667, 760
Relaxed uvula, microscopic examination of 674
Relaxation of symphysis pubis after labor 136
Relative mortality of the States 268, 340
Remarks on epithelial tumors and cancer of skin 229
Remarks on medical organization 420
" effects of iodine on the glandular system 675
Removal of foreign body from knee joint 578
Removal of a pessary after being forty-one years in pelvis 616
Reparative power of nervous system 497
Reparative power of spinal cord, after complete division 379
Report of Pennsylvania Hospital for Insane, for the year 1831. By T. S. Kirkbride, M. D., &c. bib. notice of 177
Report of committee on public hygiene 1
Report of joint committee of Philadelphia County Medical Society and Philadelphia College of Pharmacy, relative to physicians' prescriptions 50
Resolutions relative to resignation of Dr. Bell 443
Researches on reflex faculty 485
Researches, experimental, applied to pathology and physiology 481
Review of materia medica for students. By J. B. Biddle, M. D., &c. bib. notice of 109
Revolution, French 116

Rolling produced by injuries of nervous system 498
Royal Society of England 114
Royal free Hospital 744
Royal College of Physicians 738
Royal College of Surgeons 739

S.

Samuel Geo. Morton, M. D., memoir of. By C. D. Meigs, M.D. bib. notice of 109
Sanitary convention in Paris 190
Scarlet fever, lectures on. By Caspar Morris, M. D. bib. notice of 48
School of Anatomy and Medicine, adjoining St. George's Hospital 746
Senseny's history of an epidemic cholera at Chambersburg, Pa., during the summer of 1852 774
Sesquicarbonate of ammonia in lepra and psoriasis 124
Singular case of animal graft 560
Sketches of Brazil, including new views on tropical and European fever, &c. By R. Dundas, M. D. &c. bib. notice of 656
Skin, manual of diseases of, from the French of MM. Cazenave and Schedel, with notes, &c. by T. H. Burgess, M. D., and H. D. Buckley, M. D. bib. notice of 170
Softening of brain, following ligation of carotid artery 122
Sounds of heart, observations on 135
Source of vital properties 481
Spasmodic diseases, chloroform in 667
Spasmodic constriction of œsophagus 544
" asthma, case of 23
Spleen, state of, in the intermittent fever of Madagascar 545
Spread of disease and fever in Ireland 266
St. Mary's Hospital 744
St. George's " 743
St. Bartholomew's Hospital 454, 742
St. Stephen's crypt, mummy found in 185
State in which oxygen exists in the blood 613
Stomach, wound of, with protrusion 271
Stramonium, poisoning by 199
Strangulated hernia reduced by vomiting 202
Stricture dilator of Holt 611
Structure of cataract 198
Study of diseases with reference to geology 19
Substitute for mercury in syphilitic disease 198
Successful case of trephining for compression of the brain 64
Sulphate of zinc as an antiseptic 203
Sulphate of berberine 277
Surgery 64, 117, 192, 271, 547, 604
Surgical cases 218
Surgical sketches. By W. E. Horner, M. D. 753
Sydenham Society of London 110
Symes' originality 187
Symphysis pubis, relaxation of, after labor 136
Synovial articular inflammation treated with urate of ammonia 69
Syphilis, constitutional and hereditary. By E. Wilson, F. R. S., &c. bib. notice of 723
System of operative surgery, based upon the practice of surgeons in the U. S. By H. H. Smith, M. D., &c. bib. notice of 438
Syrup of orange-peel and tannin as a solvent for iron 119

T.

Tactile sensibility, central seat of 626
Taliacotian operation 341
Tannate of alumina 119
Tape-worm, cases of 629
Tartaric acid used as solvent of disulphate of quinia 204
Teeth, occasional organic union of 479
Temperature of man 554
Testimonial to Dr. Lever 59
Test for presence of mercury 614
Therapeutics 118, 204, 548, 616
Thoughts on study of diseases, with reference to geology 19
Tissues, molecular origin of 480
Tokology, theory and practice of, treatise on. By Alf. A. L. M. Velpeau, M. D., &c., translated by C. D. Meigs, M. D. With additions by W. B. Page, M. D., &c. bib. notice of 45
Topography, medical, of Lancaster county 505
Torpidity of tenrec 549
Transactions of American Medical Association, bib. notice of 93
Transactions of Medical Society of State of Pennsylvania, bib. notice of 653
Transactions of Medical Society of Virginia, bib. notice of 730
Transmission of impressions in the spinal cord 703
Transportation of two aortæ into one embryonic vertebrate 204
Treatment of gout 595

Treatise on diseases of air passages, &c. By H. Green, M. D. bib. notice of 662
Treatise on diseases of lungs and heart. By W. H. Walshe, M.D. bib. notice of 83
Trephining for compression of brain 64
Tribute to Dr. Hooker 747
" Dr. Brown-Séquard 747
Turning produced by injuries of nervous system 498
Tying of carotid artery 122

U.

Union of contiguous teeth 479
University of New York 55
" " appointments in 265
" Louisville lawsuit 268
" London 734
" College Hospital 745
Urate of ammonia in treatment of synovial articular inflammation 66
Urate of ammonia in treatment of acute metritis 531
Urethra, new instrument for cauterizing 427
Urine, albuminous, causes of 201
" microscopic characters of, in Bright's disease 466
Use of tannate of alumina 119
Uses of barium, cadmium and nickel in pharmacy 548
Uvula, relaxed, microscopic examination of 674

V.

Valentine Mott and the professorship of surgery 185
Veterinary Journal 56
Vital properties, source of 481
Voltaic pile 412

W.

Wound of stomach, with protrusion 271
Wurtz alcool entylique 749

TO THE SUBSCRIBERS OF THE "EXAMINER."

The confusion incident to the late disastrous fire in Philadelphia, in which the bindery of this Journal suffered, will, we trust, prove a sufficient apology for the delay of this number.

### NOTICE TO CORRESPONDENTS.

Communications and Books for notice should be addressed to the Editors, care of Messrs. Lindsay & Blakiston.

Letters, &c., connected with the *business affairs* of the Journal should be addressed to the Publishers.

Papers for publication must be received *before* the 20th of the month, or they cannot appear in the forthcoming number.

The following Journals have been received in exchange:

New York Medical Times.
Medical News. Philadelphia.
American Journal of Medical Sciences.
New Jersey Medical and Surgical Reporter.
New York Medical Gazette.
The Boston Medical and Surgical Journal. (Weekly.)
Western Journal.
Buffalo Journal.
Transylvania Medical Journal.
Nashville Journal of Medicine and Surgery.
Southern Medical and Surgical Journal.
Western Lancet.
North-Western Medical and Surgical Intelligencer.
Northern Lancet.
New Hampshire Journal of Medicine.
New Orleans Monthly Medical Register.
Opal, from the Utica Insane Asylum.
The London Lancet. (Weekly, London.)
The Medical Times. (Weekly, London.)
Dublin Medical Press.
Journal of Pharmacy.
North Western Medical Intelligencer.
British-American Journal of Medical and Physical Science.
American Journal of Insanity. Oct.

---

### BOOKS RECEIVED.

Walshe on the Lungs and Heart. From Messrs. Blanchard & Lea.
Hassall's Microscopic Anatomy, by Vanarsdale. American Edition.
Alin on Retro-pharyngeal Abscess.
Transactions of American Medical Association. Vol. IV.
Pereira's Materia Medica, Vol. 1, New Edition. From Blanchard & Lea.
Transactions of the College of Physicians.

---

Communications have been received from:—

Dr. T. H. Yardley, of Philadelphia.
Dr. H. Y. Smith, Philadelphia.
Dr. S. Weir Mitchell, Philadelphia.

---

The foreign correspondents of the Examiner will please direct their Exchanges and other communications to the care of Mr. Thos. Delf, No. 12 Paternoster Row, London, or Mr. H. Bossange, 21 Bis, Quai Voltaire, Paris.

